# Potential predictors for CDX2 expression loss and mismatch repair deficiency in colorectal cancer

**DOI:** 10.3389/pore.2023.1610908

**Published:** 2023-05-31

**Authors:** Ivan Vlahović, Jasmina Rajc, Ivan Švagelj, Krešimir Šolić, Dražen Švagelj

**Affiliations:** ^1^ Department of Abdominal Surgery, Clinical Hospital Center Osijek, Faculty of Medicine, University of Osijek, Osijek, Croatia; ^2^ Department of Pathology and Forensic Medicine, Clinical Hospital Center Osijek, Faculty of Medicine, University of Osijek, Osijek, Croatia; ^3^ Department of Pathology and Cytology, General County Hospital Vinkovci, Vinkovci, Croatia; ^4^ Department of Medical Statistics and Medical Informatics, Faculty of Medicine, University of Osijek, Osijek, Croatia

**Keywords:** immunohistochemistry, colorectal cancer (CRC), CDX2, dMMR, regression model

## Abstract

CDX2 expression loss is commonly associated with mismatch repair deficiency (dMMR) in colorectal cancer (CRC). However, there are only a few studies that have attempted to correlate CDX2 expression loss with specific MMR genes (*MLH1*, *MSH2*, *MSH6*, *PMS2*). This is a retrospective study of 327 patients who underwent surgery due to CRC. Nine patients (2.9%) had two synchronous CRCs, making the total sample 336 CRC. Histopathological data such as tumor type, tumor grade, perineural, lymphatic, and vascular invasion, pT stage, pN stage, peritumoral and intratumoral lymphocytic infiltration were collected and recorded in the database. After immunohistochemical analysis, CDX2 expression, MLH1, MSH2, MSH6, and PMS2 deficiency were also recorded. CDX2 expression loss was detected in 19 out of 336 CRCs (5.9%) and was associated with ascending colon CRC, partially mucinous adenocarcinoma, poorly differentiated carcinoma, and dMMR. Forty-four (13.1%) of CRCs were dMMR. We found a statistically significant association between CDX2 expression loss and MLH1 and PMS2 deficiency. Considering that most expression phenotypes include pairs of MMR genes, we analyzed MLH1/PMS2 and MSH2/MSH6 as heterodimers. Analysis of heterodimers showed a similar result, namely, that MLH1/PMS2 heterodimer deficiency was significantly associated with CDX2 expression loss. We also constructed a regression model for CDX2 expression loss and for dMMR. Poor tumor differentiation and MLH1/PMS2 heterodimer deficiency have been identified as potential predictors for CDX2 expression loss. CRC in the ascending colon and CDX2 expression loss have been identified as positive potential predictors of dMMR with rectal cancer as negative potential predictor of dMMR. Our study showed a significant association between CDX2 expression loss and MLH1 and PMS2 deficiency in CRC. We also managed to produce a regression model for CDX2 expression and showed that poor tumor differentiation and MLH1/PMS2 heterodimer deficiency are independent factors for CDX2 expression loss. We were the first to include CDX2 expression in a regression model for dMMR and showed that CDX2 expression loss can be used as a predictive factor for dMMR, which should be confirmed by further studies.

## Introduction

Colorectal cancer (CRC) is the third most common malignant tumor worldwide and the second leading cause of tumor death. It is a major public health problem, especially in developed countries [1].

CRC is treated by surgical resection, neoadjuvant therapy in some cases of rectal cancer, and adjuvant chemotherapy. Many research groups are trying to find new biomarkers that would distinguish the group of patients who benefit from adjuvant chemotherapy from the group of patients in whom chemotherapy has no effect.

One of these biomarkers is caudal homeobox 2 (CDX2). The gene encoding CDX2 is located on chromosome 13q12-13. It is a transcription factor that regulates intestinal epithelial cell differentiation. CDX2 induces differentiation and inhibits proliferation at the level of gene transcription [2]. Its expression is almost completely restricted to the gastrointestinal tract. In routine daily diagnostic practice, CDX2 is used as a marker for the identification of tumors of intestinal origin. It has been claimed that CDX2 is a tumor suppressor gene, but since it is expressed in 70%–95% of all CRC, this claim is controversial [3, 4]. Loss of CDX2 expression is associated with poor prognosis in CRC patients, as described in the publications by Dawson et al. and Bae et al. [5, 6]. Dalerba et al. analysed whether patients with CDX2 expression loss will benefit from adjuvant chemotherapy treatment. Their conclusion was that both stage II and stage III CRC patients with CDX2 expression loss might benefit from adjuvant chemotherapy. This is especially important in stage II patients who are commonly treated with surgery alone [7]. In 2022 Alarid-Escudero et al. published a cost-effectiveness analysis of CDX2 expression testing and adjuvant chemotherapy implementation for stage II colon cancer. Their conclusion was that identifying a group of stage II colon cancer patients for targeted chemotherapy based on CDX2 expression loss was a cost-effective strategy [8].

Mismatch repair system (MMR) analysis in CRC cells is part of standard pathohistological practice worldwide. *MLH1*, *MSH2*, *MSH6*, and *PMS2* are genes responsible for the mismatch repair system. A mutation in any of these genes results in mismatch repair deficiency. Immunohistochemically, CRC can be mismatch-deficient (dMMR) or mismatch-proficient (pMMR). A deficient mismatch repair system leads to microsatellite instability (MSI), which is characterized by unstable microsatellites, a type of simple DNA sequence repeat. dMMR develops in 15% of CRCs—3% in autosomal dominant Lynch syndrome and 12%–15% sporadically.

The association between CDX2 expression loss and dMMR in CRC has been described previously and is well established. In our study, we will try to find the relationship between the expression of specific MMR proteins (MLH1, MSH2, MSH6, PMS2) and CDX2 expression loss. We will also try to find potential predictors of CDX2 expression loss and dMMR.

## Materials and methods

### Patients

This is a single-institution retrospective study done at General County Hospital Vinkovci in Croatia. The patient cohort consisted of 327 patients who underwent surgery at the Department of Abdominal Surgery between 1 January 2016 and 31 December 2021. All patients underwent surgical resection for colorectal cancer. Nine patients had two synchronous tumors at the time of surgery so 336 tumor specimens were included in this study. Histopathological and immunohistochemical analysis was performed at the Department of Pathology and Cytology.

Clinical data such as age, gender, tumor location, and tumor diameter were recorded in the database. Histopathological data such as tumor type, tumor grade, perineural, lymphatic, and vascular invasion, pT stage, pN stage, peritumoral and intratumoral lymphocytic infiltration were also recorded and stored in the database. The TNM8 classification of malignant tumors was used for tumor staging [9]. Adenocarcinoma with >50% lesion composed of pools of extracellular mucin was defined as mucinous adenocarcinoma. Lesions that contain extracellular mucin but <50% were defined as partially mucinous adenocarcinoma [10]. After immunohistochemical analysis, CDX2 expression, MLH1, MSH2, MSH6, and PMS2 deficiency were also recorded. Because family history information was not available, no attempt was made to divide patients into Lynch syndrome and sporadic dMMR.

### Immunohistochemistry

Immunohistochemical analysis was performed on 4-μm sections of paraffin-embedded tissue samples. Tissue sections were incubated at 60°C for 180 min. The slides were cooled down and deparaffinized 2 min × 5 min, according to the manufacturer’s protocol with paraffin cleaning agent Tissue-Tek (Sakura, Torrance, CA, United Sates), followed by: 1 min × 5 min wash with 2-propanol, 1 min × 5 min wash with 96% ethanol, 1 min × 5 min wash with 70% ethanol and 1 × 5 min wash with deionized water. The immunohistochemical procedure was performed according to the manufacturer’s recommendations (Dako, Glostrup, Denmark). Slides were briefly prewarmed to 65°C with Dako PT Link in Target Retrieval Solution, pH 9, heated to 95°C for 20 min, and then cooled to 65°C. Slides were incubated in Washing Buffer (Dako Glostrup, Denmark), loaded into a Dako Autostainer and stained according to the standard method: Peroxidase Blocking Reagent (Dako Glostrup, Denmark), for 5 min, Washing Buffer; and primary antibodies as follows: Monoclonal mouse Anti-human CDX-2 Clone DAK-CDX2, FLEX Monoclonal Mouse Anti-Human MutL Protein Homolog 1 Clone ES05, FLEX Monoclonal Rabbit Anti-Human MutS Protein Homolog 2 Clone FE11, FLEX Monoclonal Rabbit Anti-Human MutS Protein Homolog 6 EP49, FLEX Monoclonal Rabbit Anti-Human Postmeiotic Segregation Increased 2 Clone EP51, 20 min. After washing, the slides were treated with Dako REAL EnVision/HRP for 30 min and stained with Dako REAL DAB + Chromogen. All sections were counterstained with Mayer’s hematoxylin. Slides were evaluated independently by two pathologists. The staining pattern was nuclear. One strongly positive sample of colon cancer tissue served as a positive control. Negative controls were performed by replacing the primary antibody with PBS.

**FIGURE 1:**
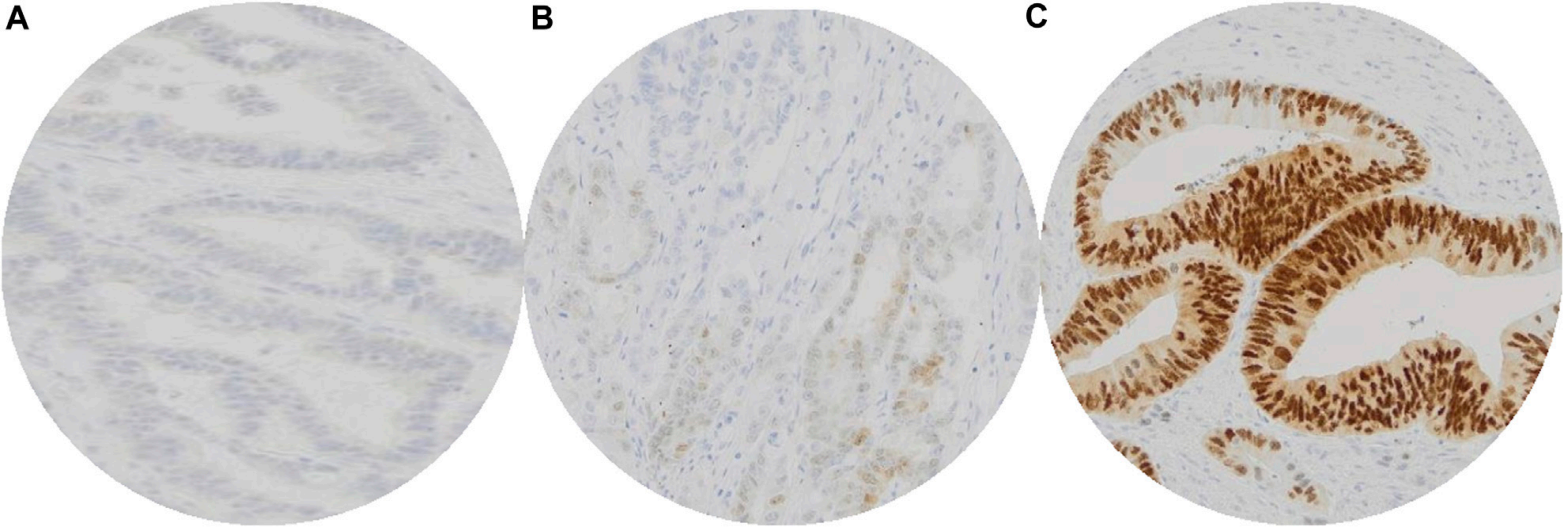
Interpretation of immunohistochemical staining of CDX2: No staining of CDX2 in colorectal carcinoma cells, valuated as score 0 **(A)**. Weak CDX2 positivity in minority (∼20%) of colorectal carcinoma cells, valuated as score 1 **(B)**. Moderate/strong CDX2 positivity of colorectal carcinoma cells, valuated as score 2 **(C)**. Tumors with scores 0 and 1 were considered negative (CDX2 expression loss) while score 2 was considered positive (normal CDX2 expression).

All immunohistochemical staining was evaluated by two independent observers (IŠ and DŠ). CDX2 expression was evaluated in accordance with Dalerba et al. and Hesteun et al. [7, 11]. Cases were divided in three groups: score 0—no staining (0%–5% positive cells), score 1—weak staining in majority of cells (5%–49% positive cells) and score 2—moderate/strong staining in a majority of cells (50%–100% positive cells). Tumors with scores 0 and 1 were considered negative (CDX2 expression loss) while score 2 was considered positive (normal CDX2 expression) (Figure 1). Weak positivity in the majority of the cells (>50%) was considered positive CDX2 staining.

CDX2 expression was immunohistochemically analysed on the same tissue samples as MMR. Independently of CDX2 expression being positive or negative the analysis was not expanded on other tissue samples of the same carcinoma so we could not exclude CDX2 expression heterogeneity.

Carcinoma was considered dMMR when there was absence of nuclear staining for at least one protein. Adjacent normal colonic epithelium, lymphocytes, and stromal cells served as positive internal controls. According to the CAP protocol for immunohistochemistry interpretation, any nuclear staining, even patchy, is taken as “no loss of expression” and only absolute absence of nuclear staining was considered “loss of expression,” provided internal controls are positive [12]. Expression of proteins was then grouped into six categories: no loss of expression, loss of expression of all four proteins, combined loss of MLH1/PMS2, combined loss of MSH2/MSH6, and isolated loss of MSH6 and PMS2.

### Statistical analysis

Standard statistical methods were used for statistical analysis. All collected categorical data were presented with absolute and relative frequencies, while numerical data were presented with arithmetic mean and standard deviation or with median and interquartile range if the distribution did not follow the Gaussian normal distribution. Results are presented in tables or graphical figures and explained in the text below. Differences between two independent sets of numerical data were tested using the parametric Student’s t-test or the nonparametric Mann-Whitney U test. Differences between categorical data were tested with Chi-Square Test or with Fisher’s Exact Test, if needed, whereas pairs of categorical data were tested with McNemar Test. In addition, Cramér’s V was calculated as a measure of association between two nominal variables when deficiency of a particular MMR gene was analyzed. Exploratory Multivariate Binary Logistic Regression was applied to the variables that were significantly different in order to identify potential predictors. Statistical analysis was performed using either MedCalc (release 19.1.3, MedCalc Software by) or IBM SPSS Statistics (release 24.0.0.0) software tools, with statistical significance defined as *α* = 0.05, where all *p* values were two-tailed.

## Results

A total of 327 patients with CRC were included (mean age was 67.6, SD = 11.6). As mentioned previously, nine patients (2.9%) had two synchronous CRCs, making the total sample 336 CRCs. Clinicopathologic characteristics are listed in Table 1. Almost two-thirds of the patients were male (Chi-Square Test, *p* = 0.004). Most tumors were pT3 and N0 according to the TNM8 classification. Tumors were most commonly located in the rectum (34.5%), were 4 cm in diameter, moderately differentiated, and lacked perineural, vascular, and lymphatic invasion. The most common tumor type was adenocarcinoma (Table 1).

**TABLE 1:** Clinicopathological variables and their association with CDX2 expression and MMR.

	Overall	CDX2−	CDX2+	*p*-value	dMMR	pMMR	*p*-value
Age/mean (SD)							
	67.6 (11.6)	69.1 (10.2)	67.5 (11.7)	0.57*	66.7 (12.2)	67.7 (11.5)	0.59*
Gender/*n* (%)							
Male	200 (61.2)	11 (5.3)	197 (94.7)	0.71^a^	25 (12.0)	183 (88.0)	0.46^a^
Female	127 (38.8)	8 (6.3)	120 (93.8)		19 (14.8)	109 (85.2)	
T status/*n* (%)							
1	8 (2.4)	0	8 (2.5)	>0.99^b^	0	8 (2.7)	0.45^b^
2	41 (12.2)	2 (10.5)	39 (12.3)		8 (18.2)	33 (11.3)	
3	273 (81.2)	17 (89.5)	256 (80.8)		34 (77.3)	239 (81.8)	
4a	4 (1.2)	0	4 (1.3)		0	4 (1.4)	
4b	10 (3.0)	0	10 (3.1)		2 (4.5)	8 (2.7)	
N status/*n* (%)							
0	189 (56.2)	13 (68.4)	176 (55.5)	0.50^b^	27 (61.4)	162 (55.5)	0.35^b^
1a	48 (14.3)	1 (5.3)	47 (14.8)		5 (11.4)	43 (14.7)	
1b	55 (16.4)	3 (15.8)	52 (16.4)		9 (20.5)	46 (15.8)	
2a	21 (6.2)	2 (10.5)	19 (6.0)		0	21 (7.2)	
2b	23 (6.8)	0	23 (7.3)		3 (6.8)	20 (6.8)	
Tumor location/*n* (%)							
A	92 (27.4)	10 (52.6)	82 (25.9)	**0.03** ^b^	33 (75.0)	59 (20.2)	**<0.001** ^b^
T	12 (3.6)	1 (5.3)	11 (3.5)		1 (2.3)	11 (3.8)	
D	48 (14.3)	4 (21.1)	44 (13.9)		1 (2.3)	47 (16.1)	
S	68 (20.2)	1 (5.3)	67 (21.1)		7 (15.9)	61 (20.9)	
R	116 (34.5)	3 (15.8)	113 (35.6)		2 (4.5)	114 (39.0)	
Tumor diameter**/**median (25%–75%)							
	4 (3–5)	4.3 (3–6.2)	4 (3–5)	0.46^c^	5 (4–6.8)	4 (3–5)	**<0.001** ^c^
Tumor type/*n* (%)							
Ac	250 (74.4)	10 (52.6)	240 (75.7)	**0.04** ^b^	18 (40.9)	232 (79.5)	**<0.001** ^a^
MAc	36 (10.7)	3 (15.8)	33 (10.4)		13 (29.5)	23 (7.9)	
PMAc	50 (14.9)	6 (31.6)	44 (13.9)		13 (29.5)	37 (12.7)	
Tumor differentiation/*n* (%)							
WD	43 (12.8)	1 (5.3)	42 (13.2)	**<0.001** ^b^	6 (13.6)	37 (12.7)	**<0.001** ^a^
MD	279 (83.0)	9 (47.4)	270 (85.2)		29 (65.9)	250 (85.6)	
PD	14 (4.2)	9 (47.4)	5 (1.6)		9 (20.5)	5 (1.7)	
Peritumoral lymphocytic infiltration/*n* (%)							
Absent	180 (53.6)	10 (52.6)	170 (53.6)	0.67^a^	16 (36.4)	164 (56.2)	**0.002** ^a^
Focal	100 (29.8)	7 (36.8)	93 (29.3)		13 (29.5)	87 (29.8)	
Present	56 (16.7)	2 (10.5)	54 (17.0)		15 (34.1)	41 (14.0)	
Intratumoral lymphocytic infiltration/*n* (%)							
Absent	125 (37.2)	8 (42.1)	117 (36.9)	0.30^a^	16 (36.4)	109 (37.3)	0.20^a^
Focal	157 (46.7)	6 (31.6)	151 (47.6)		17 (38.6)	140 (47.9)	
Present	54 (16.1)	5 (26.3)	49 (15.5)		11 (25.5)	43 (14.7)	
Vascular invasion/*n* (%)							
Absent	325 (96.7)	18 (94.7)	307 (96.8)	0.48^b^	44 (100.0)	281 (96.2)	0.37^b^
Present	11 (3.3)	1 (5.3)	10 (3.2)		0	11 (3.8)	
Lymphatic invasion/*n* (%)							
Absent	305 (90.8)	17 (89.5)	288 (90.9)	0.69^b^	41 (93.2)	264 (90.4)	0.78^b^
Present	31 (9.2)	2 (10.5)	29 (9.1)		3 (6.8)	28 (9.6)	
Perineural infiltration/*n* (%)							
Absent	276 (82.1)	15 (78.9)	261 (82.3)	0.76^b^	39 (88.6)	237 (81.2)	0.23^a^
Present	60 (17.9)	4 (21.1)	56 (17.7)		5 (11.4)	55 (18.8)	
Total	336 (100.0)	19 (100.0)	317 (100.0)		44 (100.0)	292 (100.0)	

Abbreviations: A, ascending colon; T, transverse colon; D, descending colon, S, sigmoid colon, R, rectum, Ac, adenocarcinoma; Mac, mucinous adenocarcinoma, PMAc, partially mucinous adenocarcinoma; WD, well differentiated; MD, moderately differentiated; PD, poorly differentiated.

*Student’s T-Test.

^a^ Chi-Square Test.

^b^ Fisher’s Exact Test.

^c^ Mann-Whitney U Test.

Bold values are statistically significant.

### CDX2 expression loss in relation to pathological and clinical features

CDX2 expression loss was identified in 19 out of 336 CRCs (5.9%). There was no difference in age or gender between CDX2-negative and CDX2-positive patients. Through standard statistical analysis we identified significant differences regarding CDX2 expression loss for several examined variables. CDX2 expression loss was associated with ascending colon (*p* = 0.03), partially mucinous adenocarcinoma (*p* = 0.04), poorly differentiated carcinoma (*p* < 0.001) and dMMR (Fisher’s Exact Test, *p* < 0.001). There was no statistically significant difference regarding CDX2 expression status and pT, pN, tumor diameter, peritumoral and intratumoral lymphocytic infiltration, lymphatic, vascular, and perineural invasion (Table 1).

### dMMR in relation to pathological and clinical features

dMMR was identified in 44 out of 336 CRCs (13.1%). MLH1 deficiency was found in 32 (9.1%) patients, MSH2 deficiency in nine (2.7%), MHS6 deficiency in 10 (3%), and PMS2 deficiency in 34 (10.1%) patients (Table 2). dMMR was significantly associated with ascending colon (*p* < 0.001), mucinous adenocarcinoma (*p* < 0.001) poorly differentiated carcinoma (*p* < 0.001), peritumoral lymphocytic infiltration (*p* = 0.002) and CDX2 expression loss (Fisher’s Exact Test, *p* < 0.001). Compared to pMMR, there was a statistically significant difference in tumor diameter. dMMR has, on average, 1 cm wider diameter than pMMR (Mann-Whitney U Test, *p* < 0.001). There was no statistically significant difference regarding dMMR and pT, pN, peritumoral and intratumoral lymphocytic infiltration, lymphatic, vascular, and perineural invasion (Table 1).

**TABLE 2:** Association between CXD2 expression loss and deficiency of specific MMR gene.

	CDX2−	CDX2+	*p*-value	Cramer’s V	Overall	*p*-value
MLH1/*n* (%)						
0	8 (42.1)	24 (7.6)	<0.001*	0.272	32 (9.5)	**<0.001** ^a^
1	11 (57.9)	293 (92.4)			304 (90.5)	
MSH2/*n* (%)						
0	2 (10.5)	7 (2.2)	0.09*	0.119	9 (2.7)	**<0.001** ^a^
1	17 (89.5)	310 (97.8)			327 (97.3)	
MSH6/*n* (%)						
0	2 (10.5)	8 (2.5)	0.10*	0.109	10 (3.0)	**<0.001** ^a^
1	17 (89.5)	309 (97.5)			326 (97.0)	
PMS2/*n* (%)						
0	9 (47.4)	25 (7.9)	<0.001*	0.302	34 (10.1)	**<0.001** ^a^
1	10 (52.6)	292 (92.1)			302 (89.9)	
MMR/*n* (%)						
dMMR	10 (52.6)	34 (10.7)	<0.001*	0.287	44 (13.1)	**<0.001** ^a^
pMMR	9 (47.4)	283 (89.6)			292 (86.9)	
MLH1/PMS2/*n* (%)						
0	9 (47.4)	26 (8.2)	<0.001*	0.296	35 (10.4)	**<0.001** ^a^
1	10 (52.6)	291 (91.8)			301 (89.6)	
MSH2/MSH6/*n* (%)						
0	2 (10.5)	8 (2.5)	0.104	0.109	10 (3.0)	**<0.001** ^a^
1	17 (89.5)	309 (97.5)			326 (97.0)	
Total	19 (100,0)	317 (100.0)			336 (100.0)	

*Fisher’s Exact Test.

^a^ Chi-Square Test.

Bold values are statistically significant.

### CDX2 loss in relation to MLH1, MSH2, MSH6, and PMS2

There was a statistically significant association between CDX2 expression loss and MLH1 and PMS2 deficiency (Fisher’s Exact Test, *p* < 0.001). Although statistically not significant, the association between CDX2 expression loss and MSH2 and MSH6 deficiency was four to five times higher (Table 2).

**FIGURE 2:**
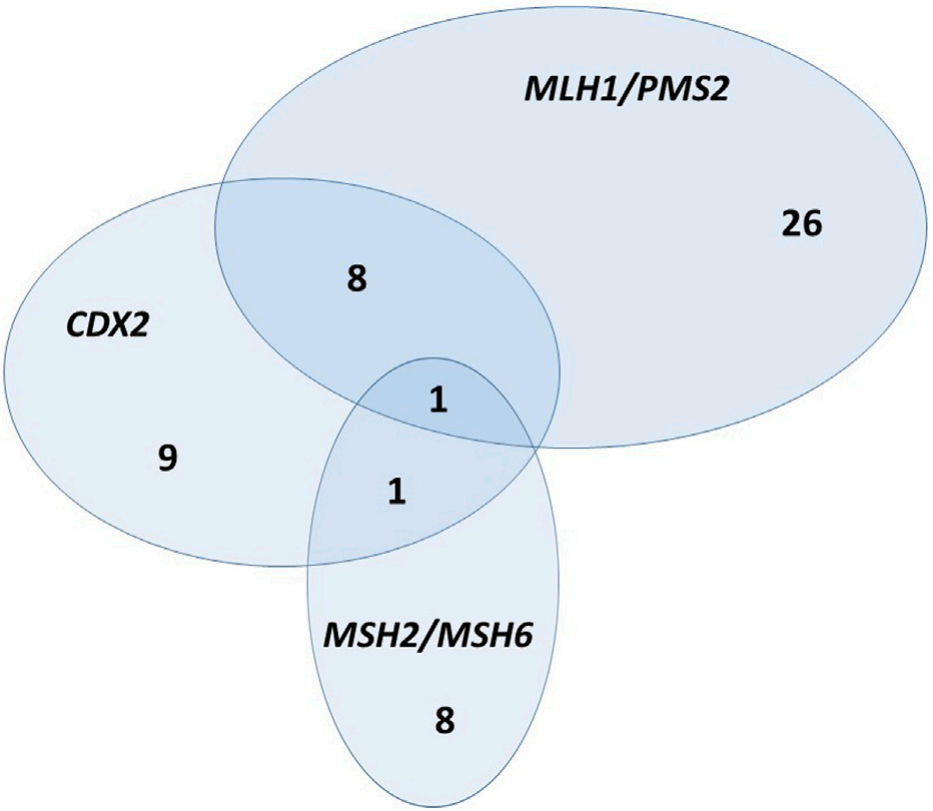
Euclid’s diagram—relationship between CDX2 expression loss, MLH1/PMS2 heterodimer, and MSH2/MSH6 heterodimer.

In our patient cohort, 44 (13.1%) CRCs were dMMR. Regarding the fact that most expression phenotypes include pairs of MMR genes, we further analyzed MLH1/PMS2 and MSH2/MSH6 as heterodimers. Analysis of heterodimers showed a similar result in the way that MLH1/PMS2 heterodimer deficiency was significantly associated with CDX2 expression loss (Fisher’s Exact Test, *p* < 0.001), while MSH2/MSH6 heterodimer was not (Figure 2). Additionally, Cramér’s V coefficient (0.296) shows the highest association when comparing MLH1/PMS2 as a heterodimer (Table 2).

### Synchronous tumors in relation to CDX2 expression loss and dMMR

Nine patients in the sample had synchronous CRC (2.9%). One patient had CDX2 expression loss and it was detected only in one of his synchronous tumors. As mentioned previously CDX2 expression loss was detected in 5.9% of tumors in the whole sample (19 out of 336) and in this group of tumors in 5.5% (1 out of 18).

Two patients had dMMR and it was detected in both of their synchronous tumors. dMMR was detected in 13.1% of tumors in the whole sample (44 out of 336) and in this group in 22.2% (4 out of 18). Results of Fisher Exact test has not shown this difference as statistically significant (*p* = 0.3). Both of the patients had the same MMR protein deficiency in their synchronous tumors. One patient had MSH2 and MSH6 deficiency in both of his synchronous tumors and the other patient had PMS2 deficiency in both of the tumors.

### Regression model for CDX2 and dMMR

The next step in statistical analysis was to make a regression model for CDX2 expression loss and dMMR. All variables that were identified as significant for CDX2 expression loss (Table 1), including MLH1/PMS2 heterodimer (Table 2) were included in the regression model.

The result was a statistically significant model (*p* < 0.001, Chi-Squared = 45.9, Cox & Snell R^2^ = 0.128, Nagelkerke R^2^ = 0.362) identifying poor tumor differentiation and MLH1/PMS2 heterodimer deficiency as potential predictors for CDX2 expression loss (Table 3) [95.83% of all cases were correctly classified (Hosmer and Lemeshow test, *p* = 0.567)].

**TABLE 3:** Potential predictors for CDX2 expression loss and dMMR.

	Coefficient	SE	Wald	p*	OR	95% CI of OR
Potential predictors for CDX2 expression loss						
CRC location—ascending colon	−0.36	0.72	0.25	0.62	0.70	0.17–2.87
Moderately differentiated CRC	0.39	1.09	0.13	0.72	1.48	0.18–12.45
Poorly differentiated CRC	4.03	1.20	11.32	**<0.001**	56.46	5.39–591.67
Adenocarcinoma	3.99	19244894.27	0.00	>0.99	53.95	
Mucinous adenocarcinoma	4.03	19244894.27	0.00	>0.99	56.29	
Partially mucinous adenocarcinoma	3.99	19244894.27	0.00	>0.99	54.53	
MLH1/PMS2 heterodimer deficiency	1.89	0.74	6.48	**0.011**	6.60	1.54–28.18
Constant	−8.03	19244894.27	0.00	>0.99		
Potential predictors for dMMR						
CRC location—ascending colon	1.17	0.48	5.91	**0.015**	3.22	1.25–8.27
CRC location—descending colon	−1.91	1.15	2.77	0.096	0.15	0.02–1.40
CRC location - rectum	−1.91	0.84	5.22	**0.022**	0.15	0.03–0.76
Adenocarcinoma	−0.63	15713390.37	0.00	>0.99	0.53	
Mucinous adenocarcinoma	0.91	15713390.37	0.00	>0.99	2.48	
Partially mucinous adenocarcinoma	0.57	15713390.37	0.00	>0.99	1.78	
Tumor diameter	0.11	0.10	1.21	0.271	1.11	0.92–1.35
Moderately differentiated CRC	0.29	0.59	0.24	0.623	1.33	0.42–4.21
Poorly differentiated CRC	1.28	1.00	1.62	0.203	3.58	0.50–25.57
Peritumoral lymphocytic infiltration—absent	−0.32	0.52	0.39	0.535	0.73	0.26–2.00
Peritumoral lymphocytic infiltration—present	0.66	0.54	1.48	0.224	1.94	0.67–5.61
CDX2 expression loss	1.85	0.84	4.89	**0.027**	6.34	1.23–32.58
Constant	−2.85	15713390.37	0.00	>0.99		

*Logistic regresion, Method: Enter.

Bold values are statistically significant.

In the regression model for dMMR, we included all variables that were identified as significant for dMMR, including CDX2 expression loss (Table 1). We got a statistically significant model (*p* < 0.001, Chi-Squard 94.1, Cox & Snell R^2^ = 0.244, Nagelkerke R^2^ = 0.453) identifying three variables as potential predictors for dMMR: CRC located in the ascending colon and CDX2 expression loss are positive potential predictors while CRC located in rectum is negative potential predictor for dMMR (Table 3) [91.37% of all cases were correctly classified (Hosmer and Lemeshow test, *p* = 0.512)].

## Discussion

CDX2 is an emerging biomarker in CRC research. In our study, CDX2 expression loss was found in 5.9% of the CRCs included in the research. According to other research groups, CDX2 expression loss ranged from 4% to 35% [3, 4, 6, 11, 13–24]. The study with the largest series of patients regarding CDX2 expression loss was performed by Dalerba et al. on 2115 patients. They found a CDX2 expression loss of 4.1%, and their result is similar to our findings [7].

In our study, CDX2 expression loss was significantly associated with CRC in ascending colon, dMMR, partially mucinous, and poorly differentiated (high grade) adenocarcinoma (Table 1). These findings are consistent with the results of other research groups. Their results showed that CDX2 expression loss was related to the right side of the colon [3–6, 11, 14, 16, 17, 19, 20, 22, 23, 25–29], dMMR [3–6, 11, 14, 16–18, 20–23, 25, 28, 29], poorly differentiated (high grade) CRC [3–5, 11, 14, 15, 17–19, 21, 23, 25] and mucinous CRC [3, 5, 6, 29]. According to the literature, some authors found an association between CDX2 expression loss and the female sex [3, 17, 18, 23, 27]. Our results show that CDX2 expression loss is not associated with age or gender, and similar results were published by Shigematsu et al, Slik et al, and Neuman et al*.* [13, 19, 22].

In 2018, Tomasello et al. published a systematic review and meta-analysis of the association of CDX2 expression with survival in early CRC. The result of their analysis was that CDX2 expression loss was negatively associated with survival [30]. Our results show that there is no association between CDX2 expression loss and the classical pathohistological features of worse survival such as high pT and pN stage, vascular, neural, and lymphatic invasion. Further research on our sample is needed to find a possible association between CDX2 expression, pathohistological features, and survival rate.

As opposed to CDX2, dMMR is an already established biomarker and an important decision factor for choosing treatment options in CRC therapy. It is present in 15% of CRCs, according to the literature, and our study shows a similar result −13.1% (Table 2). Our results show that dMMR is significantly associated with ascending colon location, mucinous adenocarcinoma, poorly differentiated carcinoma, and peritumoral lymphocytic infiltration (Table 1). These results are consistent with review articles of dMMR/MSI and CRC published by de’Angelis et al. and Boland et al*.* [31, 32].

We further analyzed individual deficiency of MMR proteins (MLH1, MSH2, MSH6, and PMS2) and their association with CDX2 expression. There is a statistically significant association between CDX2 expression loss and deficiency of MLH1 and PMS2 (*p* < 0.001) (Table 2). There is also an association between CDX2 expression loss and MSH2 and MSH6 deficiency, but it is not statistically significant (*p* = 0.09, *p* = 0.10) (Table 2).

According to the literature, there are two research groups that have studied the relationship between individual MMR protein deficiency and CDX2 expression loss in CRC. Sayar et al. published a study on 111 patients and examined only colon cancer, while rectal cancers were left out of the study. They found a strong association between CDX2 expression loss and PMS2 deficiency. All of the patients who had CDX2 expression loss had synchronous PMS2 deficiency [33]. Melincovici et al. found a similar result. In their study on 31 patients, PMS2 deficiency was significantly correlated to CDX2 expression loss, while there was no correlation between CDX2 expression loss and other MMR protein deficiency [24]. Our results are partially different from the results of these two research groups, but our sample is much bigger (336 CRCs vs. 111 and 31 CRCs).

Tóth et al. analyzed the relationship between CDX2 expression loss and individual MMR protein deficiency in CRC liver metastasis. They found that CDX2 expression loss is in a significant relationship with the deficiency of all four MMR proteins. It is hard to compare our results with theirs because of the different samples (primary CRC vs. CRC liver metastases), but it also shows the association between CDX2 expression loss and individual MMR protein deficiency [34].

The major role of the normal DNA MMR system is performed by MutSα and MutLα complexes. MutSα complex compromises MSH2/MSH6 heterodimer and MutLα MLH1/PMS2 heterodimer [35]. Therefore, we further analyzed MMR proteins as heterodimers and their relationship with CDX2 expression. As shown in Table 2, there is a statistically significant association between CDX2 expression loss and MLH1/PMS2 heterodimer deficiency, while there is no association between CDX2 expression loss and MSH2/MSH6 heterodimer deficiency. A graphical summary of this association is shown in Euclid’s diagram (Figure 2). This diagram shows that, in absolute numbers, eight CRCs have synchronous CDX2 expression loss and MLH1/PMS2 heterodimer deficiency, while only one CRC has synchronous CDX2 expression loss and MSH2/MSH6 heterodimer deficiency. One CRC has CDX2 expression loss and deficiency of both heterodimers. According to our extensive literature research, no study has analyzed the relationship between CDX2 expression and MMR heterodimer deficiency. Further research is needed to find out the possibility that the same molecular mechanism is responsible for CDX2 expression loss and MLH1/PMS2 heterodimer deficiency.

Furthermore, synchronous tumors were separated from the whole sample and analysed as a group. We found no difference in CDX2 expression loss when comparing group of synchronous tumors with the whole sample (5.9% vs. 5.5%) but the difference in more obvious, although not statistically significant (*p* = 0.3) when comparing this two groups regarding MMR (22.2% vs. 13.1%). It is likely that the larger sample size would reveal this difference as statistically significant (which should be proven in a further studies). Lee et al. made an analysis on a sample of 8,368 patients with CRC and found that 2.6% of patients had synchronous tumors which is similar to our result (2.9%) [36]. Among other CRC characteristics analysed in their study they found that patients with synchronous tumors had higher proportion of dMMR/MSI then patients with single cancer (12.8% vs. 6.6%) which is also similar to our result.

We further developed a regression model for the prediction of CDX2 expression loss and dMMR. We analyzed all variables that were significantly associated with CDX2 expression loss. Only two of them are potential predictors for CDX2 expression loss according to our regression model: poorly differentiated CRC and MLH1/PMS2 heterodimer deficiency. As CDX2 is a transcription factor that regulates intestinal epithelial cell differentiation, it is expected that poor differentiation of CRC is a predictor for CDX2 expression loss. Why the MLH1/PMS2 heterodimer deficiency is a predictor for CDX2 expression loss has to be analyzed by further molecular and (epi)genetic research. To our knowledge, this is the first published regression model for prediction of CDX2 expression loss. As mentioned in introduction Alarid-Escudero et al. published a cost-effectiveness analysis of CDX2 expression testing and adjuvant chemotherapy implementation for stage II colon cancer [8]. Based on our analysis CDX2 expression could be immunohistochemically tested only in patients with poorly differentiated CRC and MLH1/PMS2 heterodimer deficiency resulting in even less expensive immunohistochemical analysis.

All statistically significant variables according to dMMR were analyzed in the second regression model. Positive potential predictors for dMMR in CRC are CRC in ascending colon and CDX2 expression loss while CRC in rectum is negative potential predictor. According to the literature, there are five published prediction models for dMMR/MSI in CRC [37–41]. Fujiyosi et al. and Chikanati et al. made a prediction model for Japanese patients. In Asia, the incidence of dMMR/MSI is 4%–5%, in contrast to Western countries where the incidence is 10%–15%, so the sample could be different than our sample [37]. Jenkins et al. developed the “MsPath” model for patients younger than 50 years of age. Greenson et al. and Román et al. developed prediction models independent of age and with a similar population to our study sample. They found mucinous pattern to be predictor for dMMR. In our study mucinous adenocarcinoma was significantly associated with dMMR but it was not a potential predictor according to our regression model. They also found tumor infiltrating lymphocytes (TIL) to be a predictive factor. In our regression model, we did not include TIL because it was not significantly associated with dMMR in our sample. They both found Chron’s-like reaction to be a predictive factor for dMMR/MSI, but this factor was not included in our analysis, which is one of the limitations of our study. We were the first group to include CDX2 expression loss in the regression model for dMMR. We found CDX2 to be an independent prognostic factor for dMMR with a 6.34 OR. Both of these research groups found that proximal tumor location is independent prediction factor for dMMR/MSI, which is similar to our results. They divided CRC location on the right/proximal and left/distal location. In our study we divided CRC location in five categories (ascending, transversal, descending, sigmoid colon and rectum) which is more precise and found rectal cancer to be a negative potential predictor for dMMR. In 2022, Cercek et al. published that treatment of dMMR rectal cancer with anti-PD-1 monoclonal antibody Dostarlimab for 6 months resulted in complete response in all of the 12 patients included in study [41]. According to our finding that rectal cancer location is negative predictive factor for dMMR, only a minority of rectal cancer patients would be appropriate for Dostarlimab treatment and researchers would more benefit in treating proximal CRC with this cancer treatment.

We should consider the limitations of the present study. This is a single-institution study. In addition to that, we included only patients who underwent CRC resection, while patients with unresectable and uncurable tumors were left out. Another limitation of this study is the relatively low number of patients with both dMMR and CDX2 expression loss.

## Conclusion

Our study showed a significant association between CDX2 expression loss and MLH1 and PMS2 deficiency in CRC. To our knowledge, this is only the third study that compares CDX2 expression with individual mismatch protein deficiency in CRC, but with a much bigger sample than previous studies. According to our extensive literature research, we were the first to produce a regression model for CDX2 expression loss which showed that poor tumor differentiation and MLH1/PMS2 heterodimer deficiency are independent factors for CDX2 expression loss. We were also the first to include CDX2 expression in the regression model for dMMR and show that CDX2 expression loss could be used as a prediction factor for dMMR.

## Data Availability

The original contributions presented in the study are included in the article/supplementary material, further inquiries can be directed to the corresponding author.
